# Influence of specific tobacco endophytic *Bacillus* on tobacco leaf quality enhancement during fermentation

**DOI:** 10.3389/fmicb.2024.1468492

**Published:** 2024-11-25

**Authors:** Jinbin Wei, Kai Song, Zhipeng Zang, Hongjing Yang, Yuzhen Gao, Jiandong Zhang, Zhen Wang, Chen Liu

**Affiliations:** ^1^Gansu Tobacco Industry Co., Ltd., Lanzhou, Gansu, China; ^2^Technology R&D Center, Gansu Tobacco Industry Co., Ltd., Lanzhou, Gansu, China

**Keywords:** *Bacillus*, endophyte, genomics, sensory quality, tobacco fermentation

## Abstract

**Introduction:**

This study aimed to investigate the potential role of endophytic bacteria in tobacco leaves during the fermentation process to enhance the quality of tobacco.

**Methods:**

We isolated 11 endophytic bacteria from fresh tobacco leaves and selected *Bacillus halotolerans* NS36 and *Bacillus mycoides* NS75 based on sensory evaluation, both of which significantly improved the sensory quality of tobacco leaves.

**Results:**

Specifically, NS36 decreased offensive taste in tobacco leaves, while NS75 improved the quality by increasing the aroma. Chemical analysis revealed that fermentation with *B. halotolerans* NS36 significantly decreased the content of irritant compounds such as lignin, cellulose, starch, and pectin. In contrast, fermentation with *B. mycoides* NS75 reduced the content of cellulose, starch, and protein, while significantly increasing the content of Amadori compounds and glycosides. Through whole-genome sequencing, we predicted enzyme systems related to these chemical changes. *B. halotolerans* NS36 mainly secreted enzyme systems associated with the degradation of lignin, cellulose, starch, and pectin, thereby reducing irritants in tobacco leaves, diminishing unpleasant tastes, and achieving a more balanced sensory quality. *B. mycoides* NS75, on the other hand, secreted enzyme systems related to protein and glycoside hydrolysis, increasing Maillard reaction products and glycosylated compounds in tobacco leaves, thus enhancing the aroma quality and quantity.

**Discussion:**

The findings of this study offer a new perspective for the tobacco industry, namely, the use of endophytic bacilli to improve the off-flavors and aroma of tobacco leaves, which could not only enhance the industrial applicability of tobacco leaves but also potentially strengthen the market competitiveness of products. These discoveries lay the foundation for further research and application, especially in the development of new biotechnologies to improve the quality of tobacco products.

## Graphical abstract

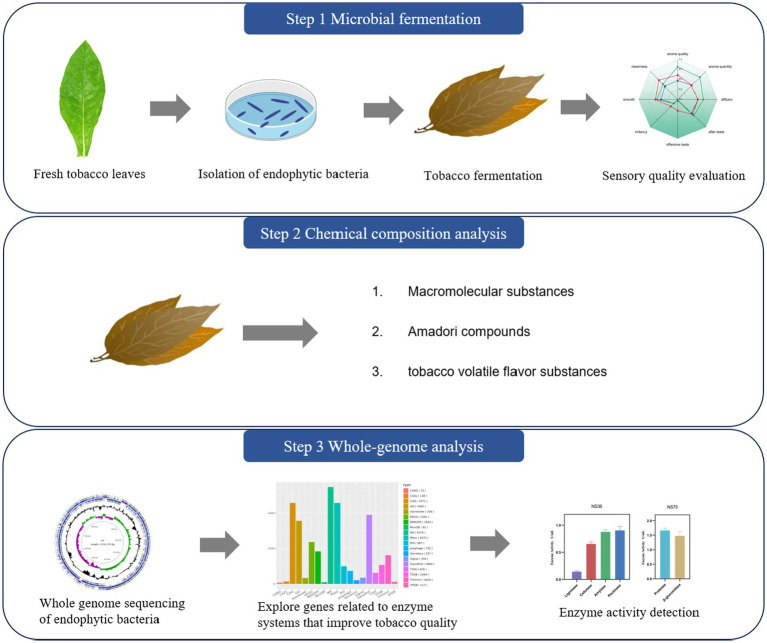

## 1 Introduction

Endophytic microorganisms colonize within plant tissues or organs, establishing a symbiotic relationship with their host plants over evolutionary time and becoming an essential component of the plant’s microenvironment (Kandel et al., 2017). As a critical cash crop, tobacco has become a focal point of research interest, with the exploration of its endophytic microbiota being vital for the sustainable development of the tobacco industry (Yan et al., 2019). The tobacco plant serves as a rich reservoir of endophytes, with over 50 distinct species identified, belonging to more than 30 genera, including *Bacillus*, *Paenibacillus*, *Pseudomonas*, and *Enterobacter* (Chen et al., 2014; Ying et al., 2015). These endophytes positively influence the physiological and metabolic processes of tobacco through their metabolic byproducts or signaling mechanisms (Ying et al., 2015).

Current research on tobacco endophytes is in its nascent stages, primarily focusing on biological control of tobacco diseases, development of biofertilizers for tobacco plants, and the degradation of harmful substances within tobacco (Zhang et al., 2017). For instance, Tao et al. found that inoculation with beneficial endophytes such as *Burkholderia* in soil can significantly enhance the tobacco defense system against pathogens and reduce disease incidence (Tao et al., 2024). Spaepen et al. demonstrated that plant growth regulators produced by endophytic *Azospirillum brasilense* can markedly promote tobacco growth (Spaepen et al., 2008). Jiang et al. isolated endophytic *Bacillus* species from tobacco leaves capable of degrading nicotine and tobacco-specific nitrosamines (Jiang et al., 2021).

*Bacillus*, known for its rapid growth, stress resistance, and biosecurity, is a dominant genus in soil and plant ecosystems (Elshaghabee et al., 2017). During tobacco fermentation, *Bacillus* significantly affects leaf quality (Hristeva et al., 2021; Michele et al., 2007; Zhao et al., 2007). Metabolites from *Bacillus subtilis* have been shown to reduce cellulose content and other macromolecules in tobacco leaves, thereby enhancing their sensory quality (Chen et al., 2024). Dominant *Bacillus* species during fermentation decrease the levels of organic and fatty acids in cigar tobacco leaves while increasing sugars and phenolic compounds, which in turn improve flavor and smoke quality (Li et al., 2023). Furthermore, an increase in key aroma compounds in tobacco leaves fermented with *Bacillus cereus* has been linked to a significant enhancement of the aroma profile (Liang et al., 2019). These findings underscore the pivotal role of *Bacillus* in tobacco fermentation processes.

However, current studies on tobacco fermentation predominantly examine *Bacillus* species on leaf surfaces, with limited research on the impact of endophytic *Bacillus* on leaf quality and their underlying mechanisms. This study focuses on two endophytic *Bacillus* strains isolated from fresh tobacco leaves, which have been shown to significantly enhance sensory quality and exhibit distinct characteristics. Using whole-genome sequencing and chemical analysis, this research elucidates the biochemical pathways through which these strains affect sensory quality, particularly focusing on their ability to increasing aroma or decreasing offensive taste during fermentation. These findings not only provide a scientific rationale for enhancing the quality of tobacco leaves and optimizing the sensory characteristics of tobacco products, but also offer valuable practical insights for the targeted application of microbes in tobacco production and processing.

## 2 Materials and methods

### 2.1 Isolation of endophytic bacteria

Following the methodology outlined by Chen et al. (2014), endophytic bacteria were isolated from mature tobacco leaves (Yuxi, Yunnan K326 variety). The leaves were collected and treated with a surface disinfection process consisting of a 10 min soak in 1% sodium hypochlorite, followed by a 2 min rinse in 75% ethanol. Sterile water rinses were performed between each step to remove residual disinfectants. The disinfected leaves were homogenized and the resulting suspension was spread onto TSA medium (containing 1.5% tryptone, 0.5% soytone, 0.5% sodium chloride, and 1.5% agar). After incubation at 28°C for 48 h, distinct colonies were selected and subcultured on TSA slants. These were then stored at 4°C for further analysis.

### 2.2 Tobacco fermentation

Tobacco leaves of the Longnan C3L-AB 2022, collected from Gansu, China, were cured and stored naturally for 2 years with an initial moisture content of 12%. Fermentation was conducted as per Zhang et al. (2024). Endophytic bacteria were cultured in LB medium (1% tryptone, 0.5% yeast extract, 1% sodium chloride) overnight to prepare the seed solution. A 1% inoculum of this solution was then transferred into TB medium (1.2% tryptone, 2.4% yeast extract, 0.94% dipotassium phosphate, 0.22% potassium dihydrogen phosphate) and incubated at 30°C with shaking at 220 rpm for 48 h Cells were collected by centrifugation at 4°C, resuspended, and sprayed onto the leaves at 1 × 10^7^ CFU/g. The inoculated leaves were fermented at 30°C and 70% humidity for 72 h. Subsequently, they were baked at 145°C for 3.5 min to mimic the cigarette production process, and then manufactured into cigarettes. Use sterile water of equal volume for spraying as a negative control CK, and use *Bacillus subtilis* YC18 isolated from the surface of tobacco leaves and proven to have quality-enhancing effects as a positive control.

### 2.3 Sensory quality assessment of tobacco samples

The Sensory quality assessment was performed by a panel of 7 professionally certified assessors, aged 25–40 years, following the standards outlined in YC/T 415–2011 for tobacco products. The assessment criteria were divided into two categories: increasing aroma (evaluated based on aroma quality, aroma quantity, diffusivity, and after taste) and decreasing offensive taste (assessed for offensive taste, irritancy, smooth, and cleanness), totaling eight distinct criteria. The evaluation took place in a controlled sensory room maintained at a temperature of 30°C and a relative humidity of 70%, ensuring optimal conditions with good ventilation. Each sensory quality attribute of the tobacco samples was rated on a 9-point scale. Scores were categorized as follows: 0–3 for poor, 4–6 for medium, and 7–9 for good. The smallest increment for scoring was 0.5 points, with higher scores reflecting superior sensory quality (Li et al., 2024).

### 2.4 Analysis of chemical components in fermented tobacco leaves

The determination of lignin, cellulose, starch, pectin, and protein content in tobacco leaves was conducted according to the method described by Jiemeng et al. (2022). The leaves were ground to a 40-mesh size, and 0.1 g of the resulting powder was homogenized with 1 mL of 80% ethanol and heated at 80°C for 30 min in a water bath. After centrifugation at 12,000 rpm for 10 min, the sediment was collected for analysis. The lignin content was measured based on the acetylation of lignin’s phenolic hydroxyl groups using the Lignin Assay Kit (G0708W, Suzhou Grace Biotechnology Co., Ltd., Suzhou, China). The starch content was determined using the iodine/potassium iodide colorimetric method with the Starch Assay Kit (G0551W, Suzhou Grace Biotechnology Co., Ltd., Suzhou, China). The pectin content was assessed using the carbazole colorimetric method with the Pectin Assay Kit (G0717W, Suzhou Grace Biotechnology Co., Ltd., Suzhou, China). The cellulose content was quantified using the anthrone colorimetric method with the Cellulose Assay Kit (G0715W, Suzhou Grace Biotechnology Co., Ltd., Suzhou, China). The protein content was determined using the bicinchoninic acid (BCA) assay with the Protein Extraction Kit (G0418W, Suzhou Grace Biotechnology Co., Ltd., Suzhou, China).

According to Liu et al. (2024), near-infrared spectroscopy was utilized to detect 17 types of Amadori compounds in tobacco leaves. The samples were air-dried to a moisture content of 6 to 8%. They were then pulverized using a mill, sifted through a 60-mesh screen, and placed into self-sealing bags from which air was evacuated and sealed. Spectral data were collected under ambient conditions of temperature (22 ± 2) °C and relative humidity (60 ± 5) %. The parameters for spectral acquisition were as follows: a scan range of 10,000–4,000 cm^−1^, a resolution of 8 cm^−1^, and 64 scans per spectrum. The integrating sphere’s light spot was positioned within two-thirds of the distance from the center of the sampling cup. Each sample’s near-infrared spectrum was collected twice, with the sample being repacked for each collection. The two spectra obtained should pass a consistency test (with a spectral similarity greater than 0.9999), and the average spectrum was taken as the sample’s near-infrared spectrum.

The determination of the aroma components in tobacco leaves, as referenced from Berenguer et al. (2021), employed a GC–MS/MS method for analysis. One gram of tobacco leaf powder was taken and mixed with 10 mL of phosphate-buffered solution (pH 3.0) and allowed to soak for 20 min. Then, 45 μL of an internal standard solution of diphenyl methanone at 0.12 mg/mL was added, followed by the addition of 10 mL of acetonitrile and vortexed for 20 min. The mixture was placed at −18°C for 30 min, then quickly supplemented with a mixture of inorganic salts (4 g MgSO_4_ and 1 g NaCl), shaken vigorously, and 5 mL of dichloromethane was added and vortexed for another 20 min. After centrifugation and standing, the supernatant was filtered through a 0.22 μm organic phase microporous membrane, and 1 mL was transferred to a chromatography vial for GC–MS/MS analysis. Chromatographic column: DB-5MS UI column (30 m × 0.25 mm × 0.25 μm, Agilent, United States). Injector temperature: 290°C. Injection volume: 1 μL. Constant flow rate: 1.5 mL/min. Temperature program: Initial temperature 40°C, held for 3 min, ramped to 75°C at 5°C/min, to 120°C at 1°C/min, to 160°C at 2°C/min, and then to 290°C at 5°C/min, held for 10 min, totaling 111 min. Ion source temperature: 280°C. Quadrupole temperature: 150°C. Scan range: 33–325 AMU. Quantitative analysis of the aroma components was conducted using the internal standard method by integrating the ion peak areas of the quantified compounds.

### 2.5 Whole genome sequencing of tobacco endophytic bacteria

Cultivate the bacterial strain to the exponential growth phase, then centrifuge the culture to pellet the bacteria. Transfer the pellet to a 1.5 mL sterile cryovial, ensuring the weight is over 1 g. Wash the pellet with sterile water to eliminate pigments from the medium. Afterward, quickly freeze the pellet in liquid nitrogen and store it at −80°C for future genomic sequencing analysis. The genomic DNA was extracted by using the Cetyltrimethyl Ammonium Bromide (CTAB) method with minor modification, and then the DNA concentration, quality and integrity were determined by using a Qubit Flurometer (Invitrogen, United States) and a NanoDrop Spectrophotometer (Thermo Scientific, United States). Sequencing libraries were generated using the TruSeq DNA Sample Preparation Kit (Illumina, United States) and the Template Prep Kit (Pacific Biosciences, United States). Genome sequencing was then performed by Personal Biotechnology Company (Shanghai, China) by using the Nanopore PromrthION48 platform/(Pacific Biosciences platform) and the Illumina Novaseq platform. Data assembly was proceeding after adapter contamination removing and data filtering by using AdapterRemoval and SOAPec (Luo et al., 2012). The filtered reads were assembled by SPAdes (Buchfink et al., 2015) and A5-miseq to constructed scaffolds and contigs. Flye and Unicycler software was used to assemble the data obtained by Nanopore platform sequencing. Subsequently, all assembled results were integrated to generate a complete sequence. Finally, the genome sequence was acquired after the rectification by using pilon software (Walker et al., 2014). Cgview was utilized to create a circular genome map (Stothard and Wishart, 2005). The hmmscan software was employed to predict the presence of CAZy enzyme-class genes within the genomic sequence (Cantarel et al., 2008). The diamond software was then used to align the sequences of protein-coding genes, which were subsequently annotated in databases such as eggNOG and KEGG (Buchfink et al., 2015).

### 2.6 Enzyme production capacity test of endophytic bacteria

Ligninase activity was measured using the ABTS method (Martin et al., 2024), with one unit defined as the change in absorbance at 1 min multiplied by 0.0278. Cellulase activity was determined using the DNS method (Suraiya et al., 2018), with one unit defined as the amount of enzyme required to produce 1 μg of glucose per mL of culture per minute. Amylase activity was assayed using the DNS method (Zhang et al., 2024), with one unit defined as the amount of enzyme required to produce 1 μg of maltose per mL of culture per minute. Pectinase activity was evaluated using the DNS method (Sharma et al., 2013), with one unit defined as the amount of enzyme required to produce 1 μg of galacturonic acid per mL of culture per minute. Protease activity was measured using the Folin-Phenol method (Li et al., 2018), with one unit defined as the amount of enzyme required to produce 1 μg of tyrosine per mL of culture per minute. *β*-glucosidase activity was determined using the pNPG method (Turner et al., 2002), with one unit defined as the amount of enzyme required to produce 1 μM of pNP per mL of culture per minute.

## 3 Results

### 3.1 Isolation and identification of endophytic bacteria in tobacco and impact on the sensory quality assessment of tobacco leaves

**Figure 1:**
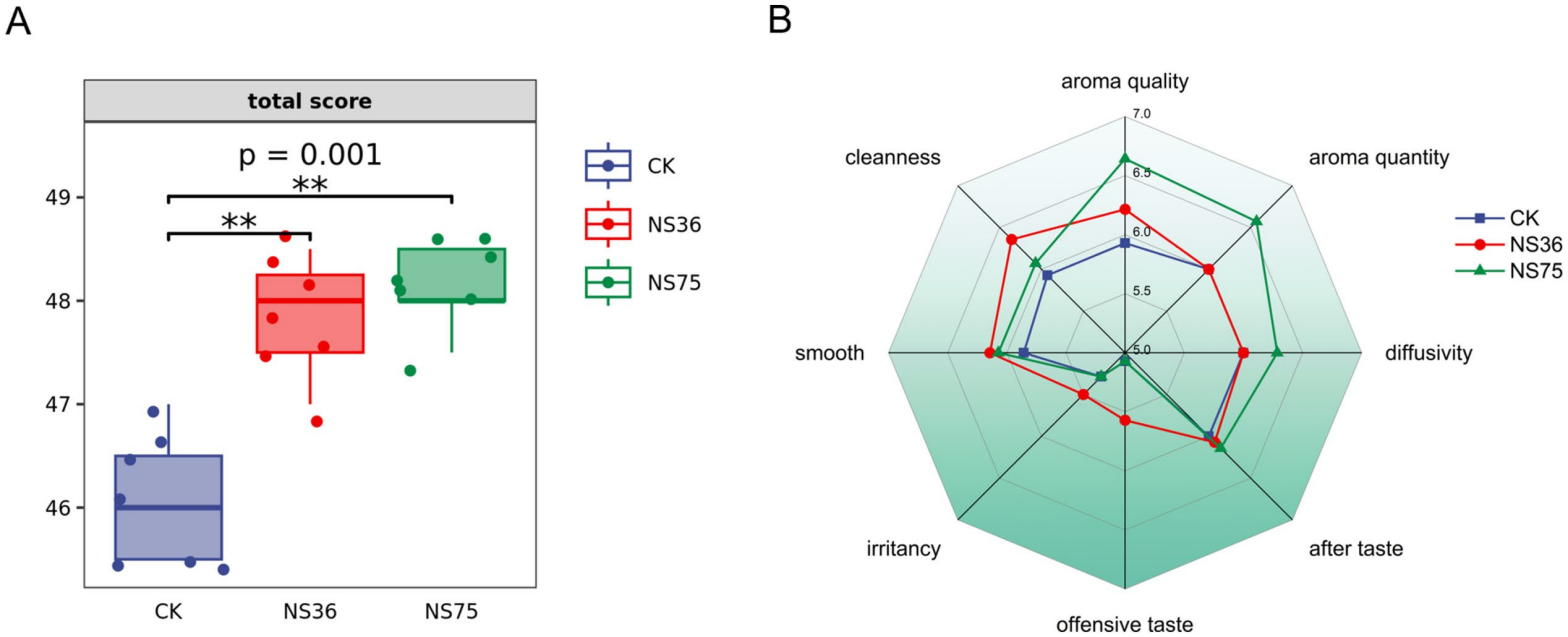
Sensory quality assessment of tobacco samples. Using tobacco leaves treated with sterile water as CK. **(A)** Sensory quality assessment total scores of tobacco leaves treated by two strains of endophytic bacteria. **(B)** According to the sensory quality assessment criteria for tobacco in-process products, the assessment is conducted based on eight assessment indicators.

This study isolated 11 strains of endophytic bacteria from fresh tobacco leaves and conducted a sensory quality assessment of the fermented tobacco leaves. As shown in Supplementary Table S1, endophytic bacteria strains NS36 and NS75 demonstrated significant effects in enhancing the quality of tobacco leaves. The fermented tobacco leaves showed a significant increase in the total sensory quality assessment score (Figure 1A), but they differed in their contributions to the improvement of tobacco quality. Strain NS36 primarily decreased offensive taste of the tobacco leaves, while NS75 significantly increasing the aroma of the tobacco leaves (Figure 1B).

### 3.2 Analysis of the chemical composition of tobacco leaves after fermentation by endophytes

**Figure 2:**
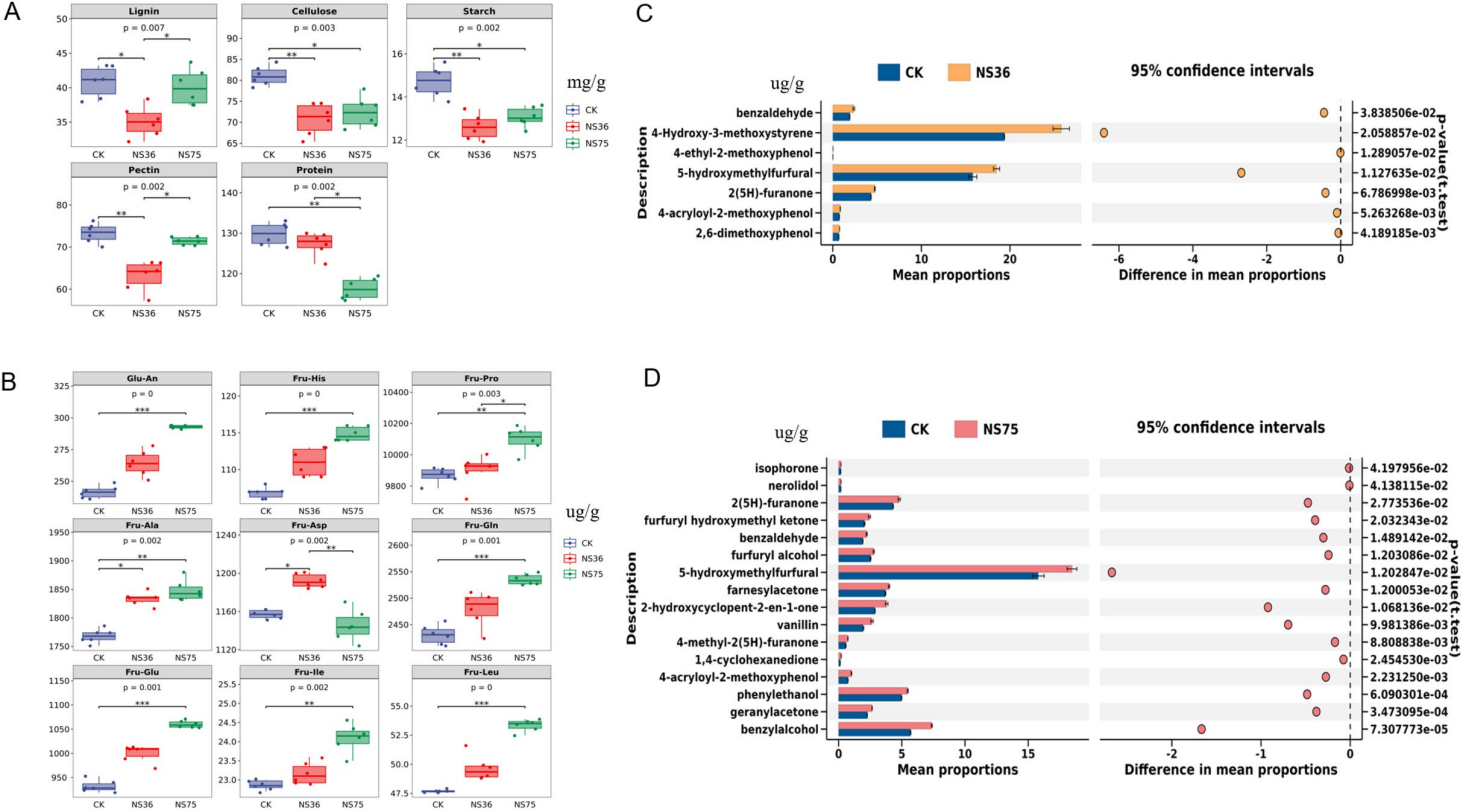
Chemical composition analysis of tobacco leaves after endophytic bacteria fermentation. Using tobacco leaves treated with sterile water as CK. **(A)** Lignin, cellulose, starch, pectin, and protein content in tobacco leaves. **(B)** The content of 17 Amadori compounds in tobacco leaves, Glu-An: Glucosamine; Fru-His: N-(1-deoxy-*β*-d-fructopyranos-1-yl)-l-histidine; Fru-Pro: N-(1-deoxy-β-d-fructopyranos-1-yl)-l-proline; Fru-Ala: N-(1-deoxy-β-d-fructopyranos-1-yl)-l-alanine; Fru-Asp: N-(1-deoxy-β-d-fructopyranos-1-yl)-l-aspartic acid; Fru-Gln: N-(1-deoxy-β-d-fructopyranos-1-yl)-l-glutamine; Fru-Glu: N-(1-deoxy-β-d-fructopyranos-1-yl)-l-glutamic acid; Fru-Ile: N-(1-deoxy-β-d-fructopyranos-1-yl)-l-isoleucine; Fru-Leu: N-(1-deoxy-β-d-fructopyranos-1-yl)-l-leucine. **(C,D)** Detection of flavor components in fermented tobacco leaves were conducted using GC–MS/MS, and differential flavor components were visualized with the STAMP software, with a significance level of *p* < 0.05.

By comparing the content of macromolecular substances in tobacco leaves before and after fermentation by the two endophytic bacteria, we found that the content of lignin, cellulose, starch, and pectin in tobacco leaves fermented by NS36 was significantly reduced, while the content of cellulose, starch, and protein in tobacco leaves fermented by NS75 was significantly reduced (Figure 2A). Near-infrared spectroscopy was used to detect 17 Amadori compounds (Supplementary Table S2), and the results showed that the content of Fru-Ala and Fru-Asp in tobacco leaves fermented by NS36 was significantly increased, while the content of Glu-An, Fru-His, Fru-Pro, Fru-Ala, Fru-Gln, Fru-Glu, Fru-Ile, and Fru-Leu in tobacco leaves fermented by NS75 was significantly increased (Figure 2B). Using GC–MS/MS technology to detect 55 volatile flavor compounds in fermented tobacco leaves (Supplementary Table S3), and performing inter-group difference analysis with stamp software, the results showed that the content of benzylalcohol, 4-Hydroxy-3-methoxystyrene, 4-ethyl-2-methoxyphenol, 5-hydroxymethylfurfural, 2(5H)-furanone, 4-acryloyl-2-methoxyphenol, and 2,6-dimethoxyphenol in tobacco leaves fermented by NS36 was significantly increased (Figure 2C), while the content of isophorone, nerolidol, 2(5H)-furanone, furfuryl hydroxymethyl ketone, benzaldehyde, furfuryl alcohol, 5-hydroxymethylfurfural, farnesylacetone, 2-hydroxycyclopent-2-en-1-one, vanillin, 4-methyl-2(5H)-furanone, 1,4-cyclohexanedione, 4-acryloyl-2-methoxyphenol, phenylethanol, geranylacetone, and benzylalcohol in tobacco leaves fermented by NS75 was significantly increased (Figure 2D).

### 3.3 Whole genome sequencing of tobacco endophytes

**Figure 3:**
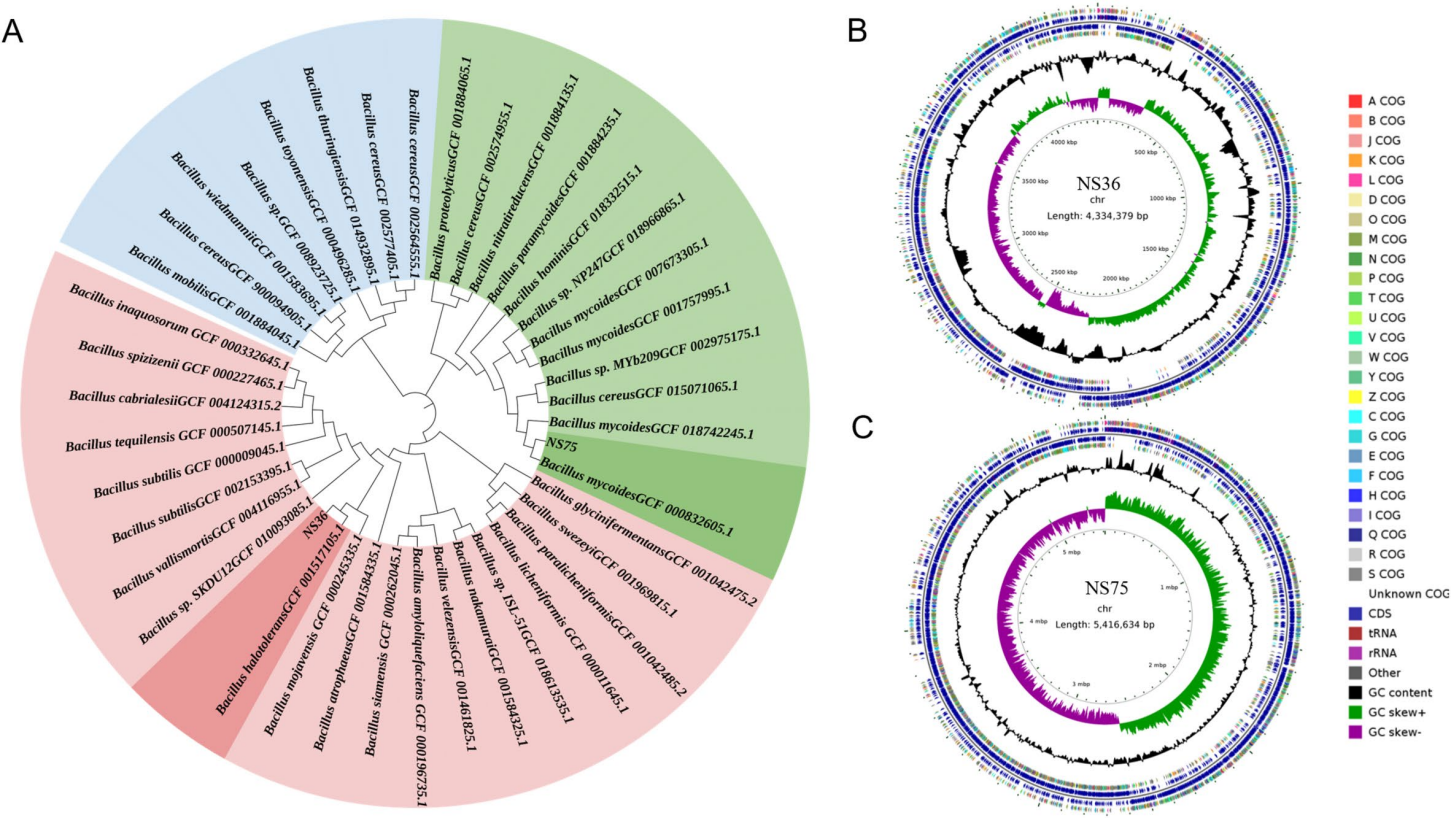
Whole genome sequencing of endophytic bacteria. **(A)** Based on the chromosomal sequences from the assembly results, construct a phylogenetic tree for the core genes of the sample. **(B,C)** Endophyte genome circular map, from the inside out: the first circle represents the scale; the second circle represents GC Skew; the third circle represents GC content; the fourth and seventh circles represent each CDS belonging to COG; the fifth and sixth circles represent the positions of CDS, tRNA, and rRNA on the genome.

By sequencing the genomes of the two endophytic bacteria strains NS36 and NS75 using the Illumina NovaSeq combined with the PacBio Sequel platform, we identified closely related species in databases using the fastANI software based on the chromosomal sequences from the assembly results. We also constructed a phylogenetic tree of core genes using the MEGA-X software, which showed that NS36 is *Bacillus halotolerans* and NS75 is *Bacillus mycoides* (Figure 3A). The genome of *B. halotolerans* NS36 consists of a complete chromosome of 4,334,379 bp with a GC content of 43.40%, encoding 4,470 protein-coding genes, of which 940 are core genes, accounting for 21.02% (Figure 3B; Table 1). The genome of *B. mycoides* NS75 consists of a complete chromosome of 5,416,634 bp with a GC content of 35.51%, encoding 5,493 protein-coding genes, of which 1,006 are core genes, accounting for 17.33% (Figure 3C; Table 1).

**Table 1:** Statistical analysis of basic genomic information.

Sample	NS36	NS75
Genome size (bp)	4,334,379	5,416,634
G + C (%)	43.4	35.51
tRNA genes	86	107
rRNA genes	30	42
CRISPRs	2	0
Protein-coding genes	4,470	5,493
Protein-coding	87.36%	82.93%
CoreGenes	940 (21.02%)	1,006 (17.33%)
DispensableGenes	3,530	4,797

### 3.4 Analysis of the mechanism of tobacco fermentation by endophytes

**Figure 4:**
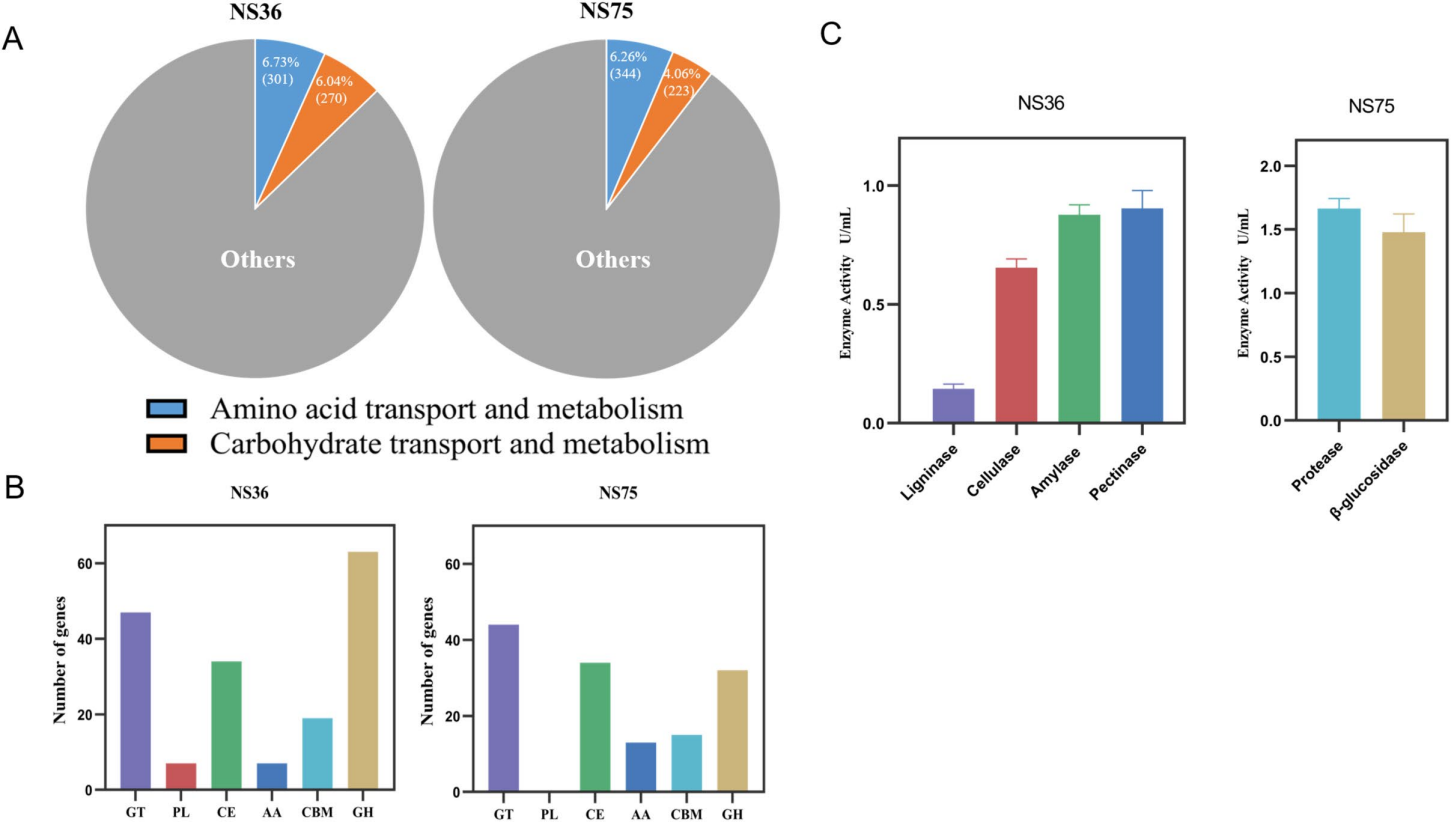
Analysis of the mechanism of endophytic bacteria fermentation in tobacco leaves. **(A)** The proportions of “amino acid transport and metabolism” and “carbohydrate transport and metabolism” in the eggNOG classification. **(B)** CAZy Functional Classification. **(C)** *In vitro* enzyme activity assay.

Based on the functional classification analysis from the eggNOG database, the predicted protein-coding genes were categorized into various biological processes and functional categories (Supplementary Figure S1). In both the “Amino acid transport and metabolism” category and the “Carbohydrate transport and metabolism” category, the proportion of the two strains accounted for more than 10% (Figure 4A).

Whole-genome analysis of *B. halotolerans* NS36 revealed 177 genes belonging to the CAZymes family, with the highest number of Glycoside Hydrolases, Glycosyl Transferases, and Carbohydrate Esterases, followed by Carbohydrate-Binding Modules, Auxiliary Activities, and Polysaccharide Lyases (Figure 4B). Table 2 illustrates the distribution of enzymes related to the degradation of Lignin, Cellulose, Starch, and Pectin within the NS36 genome sequence. Specifically, we identified 5 genes associated with Lignin degradation, including Laccase (EC 1.10.3.2), Vanillyl alcohol oxidases (EC 1.1.3.38), Benzoquinone reductases (EC 1.6.5.6), and Lytic polysaccharide monooxygenases (EC 1.14.99.54). In terms of Cellulose degradation, we characterized 9 related genes, including Exocellulase (EC 3.2.1.74), *β*-glucosidases (EC 3.2.1.21), and Endo-β-1,4-glucanase (EC 3.2.1.4, EC 3.2.1.91). There were 20 genes related to Starch degradation, including Alpha-amylase (EC 3.2.1.1), Beta-amylase (EC 3.2.1.2), Glucoamylase (EC 3.2.1.3), Alpha-glucosidase (EC 3.2.1.10). For Pectin degradation, 26 genes were retrieved, including Exo-polygalacturonase (EC 3.2.1.67), Polygalacturonase (EC 3.2.1.15), Rhamnogalacturonan lyase (EC 4.2.2.-), Pectin lyase (EC 4.2.2.10), Pectin lyase (EC 4.2.2.2), Pectate disaccharide-lyase (EC 4.2.2.9), Rhamnogalacturonan endolyase (EC 4.2.2.23), Polysaccharide Lyases (EC 4.2.2.9), and Pectin acetylesterase (EC 3.1.1.6). These findings indicate that *B. halotolerans* NS36 has the capability to degrade complex macromolecular compounds in tobacco, which may be a key mechanism for its ability to decrease the offensive taste.

**Table 2:** Distribution of enzymes related to lignin, cellulose, pectin, and starch degradation in the NS36 genome sequence.

Macromolecules	Enzymes	Distribution in CAZy database
Lignin	Laccase (EC 1.10.3.2)	AA1 (chr_904)
	Vanillyl alcohol oxidases (EC 1.1.3.38)	AA4 (chr_3333)
	Benzoquinone reductases (EC 1.6.5.6)	AA6 (chr_1211, chr_1242)
	Lytic polysaccharide monooxygenases (EC 1.14.99.54)	AA10 (chr_2295)
Cellulose	Exocellulase (EC 3.2.1.74)	GH1 (chr_3883, chr_3902, chr_4323, chr_587, chr_859)
	β-glucosidases (EC 3.2.1.21)	GH3 (chr_406)
	Endo-β-1,4-glucanase (EC 3.2.1.4, EC 3.2.1.91)	GH5 (chr_2217), GH51 (chr_3316, chr_3340)
Starch	Alpha-amylase (EC 3.2.1.1)	GH13 (chr_542, chr_518, chr_3599, chr_3562, chr_3466, chr_2096, chr_1065), GH16 (chr_2152), GH18 (chr_132, chr_1719, chr_18, chr_848), GH43 (chr_2221)
	Beta-amylase (EC 3.2.1.2)	GH42 (chr_143, chr_992), GH68 (chr_178)
	Glucoamylase (EC 3.2.1.3)	GH26 (chr_863), GH120 (chr_2478)
	Alpha-glucosidase (EC 3.2.1.10)	GH76 (chr_4443)
Pectin	Exo-polygalacturonase (EC 3.2.1.67)	GH4 (chr_1121, chr_3501, chr_3953, chr_997)
	Polygalacturonase (EC 3.2.1.15)	GH16 (chr_2152), GH32 (chr_179, chr_3160, chr_4009)
	Rhamnogalacturonan lyase (EC 4.2.2.-)	GH23 (chr_1478, chr_1601, chr_2338, chr_2565, chr_3035, chr_4419, chr_755)
	Pectin lyase (EC 4.2.2.10)	PL1 (chr_2272, chr_1037)
	Pectin lyase (EC 4.2.2.2)	PL3 (chr_222)
	Pectate disaccharide-lyase (EC 4.2.2.9)	PL9 (chr_4184)
	Rhamnogalacturonan endolyase (EC 4.2.2.23)	PL11 (chr_988, chr_989)
	Polysaccharide Lyases (EC 4.2.2.9)	PL26 (chr_993)
	Pectin acetylesterase (EC 3.1.1.6)	CE12 (chr_3004, chr_3896, chr_985, chr_991)

The CAZymes family in *B. mycoides* NS75 includes 32 genes belonging to the Glycoside Hydrolases family (Figure 4B). Specifically, we identified 6 beta-glucosidases (EC 3.2.1.21), 1 alpha-galactosidase (EC 3.2.1.22), 9 alpha-amylases (EC 3.2.1.1), 3 chitinases (EC 3.2.1.14), 3 peptidoglycan lyases (EC 4.2.2.-), 3 peptidoglycan hydrolases (EC 3.2.1.-), 3 alpha-N-acetylgalactosaminidases (EC 3.2.1.49), 1 beta-mannanase (EC 3.2.1.78), and 1 endo-alpha-1,4-polygalactosaminidase (EC 3.2.1.109) (Table 3). Further analysis of NS75’s whole-genome data for secondary metabolic pathways through the KEGG database (Supplementary Figure S2) identified 28 genes directly related to protein degradation, which are involved in Carbohydrate metabolism, Folding, sorting and degradation, Glycan biosynthesis and metabolism, Metabolism of other amino acids, and Protein families: metabolism (Table 4). The activity of these genes not only promotes the efficient degradation of proteins in tobacco leaves but also releases amino acids and other small molecules that enhance the aroma, thereby improving the overall aroma quality of the tobacco leaves.

**Table 3:** Distribution of glycoside hydrolase enzymes in the NS75 genome sequence.

Enzymes	Distribution in CAZy database
Beta-glucosidase (EC 3.2.1.21)	GH1 (chr_3803, chr_917, chr_1175, chr_1930, chr_3697), GH3 (chr_186)
Alpha-galactosidase (EC 3.2.1.22)	GH4 (chr_5204)
Alpha-amylase (EC 3.2.1.1)	GH13 (chr_1129, chr_4738, chr_2502, chr_4056, chr_599, chr_398, chr_4057, chr_3465, chr_4908)
Chitinase (EC 3.2.1.14)	GH18 (chr_3567, chr_3762, chr_413)
Peptidoglycan lyase (EC 4.2.2.-)	GH23 (chr_1658, chr_2400, chr_3398)
Peptidoglycan hydrolase (EC 3.2.1.-)	GH74 (chr_1850, chr_3272, chr_3516)
Alpha-N-acetylgalactosaminidase (EC 3.2.1.49)	GH109 (chr_3252, chr_3556, chr_4706)
Beta-mannanase (EC 3.2.1.78)	GH113 (chr_3167)
Endo-alpha-1,4-polygalactosaminidase (EC 3.2.1.109)	GH114 (chr_5449)

**Table 4:** Annotation table of protease genes in NS75.

Gene name	Gene description	Enzyme	Gene ID
*mapP* (K06896)	Carbohydrate metabolism	Maltose 6′-phosphate phosphatase	chr_400
*lepB* (K03100)	Folding, sorting and degradation	Signal peptidase I	chr_442, chr_1108, chr_3016, chr_3028, chr_3879
*lspA* (K03101)	Folding, sorting and degradation	Signal peptidase II	chr_3934
*dacC* (K07258)	Glycan biosynthesis and metabolism	Serine-type D-Ala-D-Ala carboxypeptidase	chr_4121
*pepA* (K01255)	Metabolism of other amino acids	Leucyl aminopeptidase	chr_4942
*clpP* (K01358)	Protein families: metabolism	ATP-dependent Clp protease	chr_2736, chr_5148
*ctpA* (K03797)	Protein families: metabolism	Carboxyl-terminal processing protease	chr_5180
*erfK* (K16291)	Protein families: metabolism	L,D-transpeptidase ErfK/SrfK	chr_727
*iadA* (K01305)	Protein families: metabolism	Beta-aspartyl-dipeptidase (metallo-type)	chr_3239
*ldcA* (K01297)	Protein families: metabolism	Muramoyltetrapeptide carboxypeptidase	chr_1352
*lon* (K01338)	Protein families: metabolism	ATP-dependent Lon protease	chr_4511, chr_4512
*map* (K01265)	Protein families: metabolism	Methionyl aminopeptidase	chr_137, chr_1555, chr_5364
*mep* (K19303)	Protein families: metabolism	Murein DD-endopeptidase	chr_1913, chr_1417
*pepD* (K01270)	Protein families: metabolism	Dipeptidase D	chr_2383
*pepF* (K08602)	Protein families: metabolism	Oligoendopeptidase F	chr_1182
*pepP* (K01262)	Protein families: metabolism	Xaa-Pro aminopeptidase	chr_1851, chr_4248
*pepT* (K01258)	Protein families: metabolism	Tripeptide aminopeptidase	chr_3784
*uppS* (K00806)	Protein families: metabolism	Undecaprenyl diphosphate synthase	chr_3863

In terms of enzyme activity analysis, *B. halotolerans* NS36 demonstrated the activity of ligninases, cellulases, amylases, and pectinases, while *B. mycoides* NS75 exhibited protease and *β*-glucosidase activities (Figure 4C). The enzymatic activities exhibited by these two endophytic bacteria are consistent with the results of their whole-genome analysis, revealing their potential significant roles in the tobacco fermentation process. These findings provide important molecular mechanisms for further exploration of the application of endophytic bacteria in tobacco fermentation.

## 4 Discussion

This study isolated 11 strains of tobacco endophytic bacteria from fresh tobacco leaves and conducted sensory quality assessment of their roles in the tobacco fermentation process (Supplementary Table S1). The results indicated that the endophytic bacteria *B. halotolerans* NS36 and *B. mycoides* NS75 significantly enhanced the sensory quality of tobacco leaves (Figure 1A). Notably, these two strains exhibited different characteristics in improving tobacco quality (Figure 1B). *B. halotolerans* NS36 primarily decreased offensive taste in tobacco leaves, while *B. mycoides* NS75 significantly increased the aroma of the tobacco leaves. To further verify the specific impact of these two endophytic bacteria on tobacco quality, we conducted a detailed chemical analysis of the fermented tobacco leaves in terms of macromolecular substances, Amadori compounds, and tobacco volatile flavor substances. The results showed (Figure 2) that there were significant differences in the chemical composition of tobacco leaves fermented by NS36 and NS75, which may be the cause of different sensory experiences.

After fermentation by *B. halotolerans* NS36, the levels of lignin, cellulose, starch, and pectin, which are irritant compounds, were reduced (Figure 2A), and the content of phenolic substances such as 4-Hydroxy-3-methoxystyrene, 4-ethyl-2-methoxyphenol, 4-acryloyl-2-methoxyphenol, and 2,6-dimethoxyphenol significantly increased (Figure 2C). The difficult-to-degrade macromolecular substances in tobacco leaves include lignin, cellulose, starch, and pectin, which are the main sources of irritation and off-flavors during sensory assessment (Chen et al., 2019). Lignin, as a major component of tobacco stems, excessive presence can lead to a strong woody odor in tobacco leaves and a burning throat sensation (Yang et al., 2024). Cellulose is crucial for maintaining the structure and combustion stability of tobacco leaves, but an excess of cellulose weakens the plasticity of tobacco leaves, increases irritation, and affects sensory quality (Kong et al., 2024). Control of starch content is equally important as it directly affects the combustibility of tobacco leaves and the irritant gases produced during the smoking process (Dong et al., 2015; Gao et al., 2006). Pectin plays a key role in the moisture retention and flexibility of tobacco leaves; however, an excessive amount of pectin can produce methanol during combustion, thereby affecting sensory quality (Bai et al., 2015). Furthermore, the content of phenolic substances such as 4-Hydroxy-3-methoxystyrene, 4-ethyl-2-methoxyphenol, 4-acryloyl-2-methoxyphenol, and 2,6-dimethoxyphenol in tobacco leaves fermented by NS36 significantly increased (Figure 2C), a phenomenon that may be due to the degradation of lignin (Adelakun et al., 2012; Yuan et al., 2022). Therefore, these findings suggest that *B. halotolerans* NS36 has the ability to degrade irritant macromolecular compounds in tobacco, which may be the key reason for its improvement of tobacco off-flavors and irritation.

In contrast, the content of cellulose, starch, and protein in tobacco leaves fermented by *B. mycoides* NS75 was significantly reduced (Figure 2A), and the content of Amadori compounds such as Glu-An, Fru-His, and Fru-Pro significantly increased (Figure 2B). The Maillard reaction is a browning reaction that occurs between reducing sugars or carbonyl compounds and compounds containing free amino acids at room temperature or upon heating (Feng et al., 2024). Through the fermentation of *B. mycoides* NS75, cellulose and starch in tobacco leaves are degraded into reducing sugars, and proteins are degraded into amino acids. The continuous Maillard reaction between reducing sugars and amino acids generates compounds with characteristic fragrance, thereby increasing the aroma quality and quantity of tobacco leaves. Amadori compounds are intermediates of the Maillard reaction formed by the condensation of amino acids with monosaccharides, which can increase the sweet and baked fragrance of tobacco leaves. The increase in Amadori compounds after the fermentation of tobacco leaves by *B. mycoides* NS75 (Figure 2B) further confirms the impact of the Maillard reaction on the sensory quality of tobacco leaves. At the same time, amino acids can also generate a series of fragrant substances such as benzaldehyde, phenylacetaldehyde, phenylmethanol, and phenylethanol through high-temperature pyrolysis (Yin et al., 2022). In addition, a considerable part of the flavor components in tobacco leaves exists in the form of glycosides. Studies have shown that after the glycosidic substances in tobacco leaves are hydrolyzed by *β*-glycosidase, aglycones such as 3-methylbutanol, phenylmethanol, and 4-hydroxyequol are released, indicating that the glycosidic aroma precursors in tobacco leaves are not completely pyrolyzed during the combustion process, and the comprehensive utilization rate is low (Kodama et al., 1981). After the fermentation of tobacco leaves by *B. mycoides* NS75, the content of glycosidic aglycones such as isophorone, nerolidol, and 2(5H)-furanone significantly increased (Figure 2D), and the increase in these substances also enhanced the aroma quantity and aroma quality of tobacco leaves.

Tobacco leaf fermentation is a complex biochemical process in which microorganisms play a key role in shortening the fermentation period, enhancing tobacco quality, reducing harmful substances, and increasing safety (Jiemeng et al., 2022; Zhang et al., 2024). Current research trends show that the enzymatic systems produced by functional microorganisms during metabolism can promote the degradation of macromolecular substances, thereby increasing the concentration of small molecules of aromatic substances in tobacco leaves and reducing irritation (Zhang et al., 2024). Chemical analysis found that *B. halotolerans* NS36 fermented tobacco leaves could reduce the content of irritant compounds such as lignin, cellulose, starch, and pectin, while *B. mycoides* NS75 fermented tobacco leaves could increase the content of volatile substances such as Amadori compounds and glycosidic aglycones. Therefore, this study focused on genes related to these enzymatic systems through whole-genome sequencing. According to the functional classification analysis of the eggNOG database, the predicted protein-coding genes were classified into different biological processes and functional categories (Supplementary Figure S1). In the “Amino acid transport and metabolism” category and the “Carbohydrate transport and metabolism” category, the proportion of the two strains was more than 10%, revealing that these two endophytic bacteria contain a large number of genes involved in the metabolism of amino acids and carbohydrates, which may play a key role in the fermentation of tobacco leaves (Figure 4A).

*B. halotolerans* NS36 has 177 genes belonging to the CAZymes family, with the highest number of Glycoside Hydrolases, Glycosyl Transferases, and Carbohydrate Esterases, followed by Carbohydrate-Binding Modules, Auxiliary Activities, and Polysaccharide Lyases (Figure 4B). These genes may play an important role in the degradation of irritant substances including lignin, cellulose, starch, and pectin. For example, AA1, AA4, AA6, and AA10 families may be related to lignin degradation, GH1, GH3, GH5, and GH51 families are related to cellulose degradation, GH13, GH18, GH26, GH42, GH43, and GH76 families may be related to starch degradation, and GH4, GH16, GH23, PL1, PL3, PL9, PL11, and CE12 are related to pectin degradation (Jiemeng et al., 2022). By searching, we found some enzyme genes related to the degradation of irritant substances (Table 2), including 5 genes related to lignin degradation, 9 genes related to cellulose degradation, 20 genes related to starch degradation, and 26 genes related to pectin degradation. These genes may play a key role in the degradation of lignin, cellulose, pectin, and starch in tobacco leaves. For instance, Laccase (EC 1.10.3.2) in the degradation process of tobacco lignin catalyzes the oxidation of phenolic monomers in lignin, producing active free radical intermediates, leading to the modification and decomposition of the lignin structure, thereby reducing off-flavors and irritation in tobacco leaves and enhancing the sensory quality of tobacco (Strong and Claus, 2011). Exocellulase (EC 3.2.1.74) mainly acts on the terminal of cellulose polysaccharide chains, releasing cellobiose and glucose, and is one of the key enzymes in the cellulose degradation process (Bao et al., 2011). Alpha-amylase (EC 3.2.1.1) can act on the *α*-1,4 glycosidic bonds within the starch molecule, thereby breaking down starch into small molecules such as dextrin, oligosaccharides, and monosaccharides such as glucose and maltose (Farias et al., 2021). Rhamnogalacturonan lyase (EC 4.2.2.-) can specifically recognize and cut the α-1,4 glycosidic bonds on the main chain of rhamnogalacturonan I (RG-I) in tobacco pectin, leading to the degradation of pectin in tobacco leaves (Ochoa and Tiznado, 2018).

*B. mycoides* NS75 has 138 genes belonging to the CAZymes family, of which 32 genes are classified as Glycoside Hydrolases (Figure 4B). These genes may play an important role in hydrolyzing glycosides in tobacco and releasing aglycones (Table 3). For example, beta-glucosidase (EC 3.2.1.21) in the GH1 and GH3 families can hydrolyze glycosidic bonds, converting flavor precursors into aromatic substances with rich aroma, thereby playing a role in enhancing aroma (Muradova et al., 2023). Alpha-galactosidase (EC 3.2.1.22) in the GH4 family can catalyze the hydrolysis of α-D-galactoside bonds, releasing galactose from various substrates, including polysaccharides, glycolipids, and glycoproteins (Katrolia et al., 2014). These findings indicate that *B. mycoides* NS75 hydrolyzes glycosides in tobacco leaves through glycosidase hydrolysis, significantly increasing the amount of a series of tobacco glycoside conjugates, thereby enhancing the flavor components in tobacco. In addition, through the KEGG database analysis of *B. mycoides* NS75’s whole-genome data for secondary metabolic pathways (Supplementary Figure S2), we identified 28 genes directly related to protein degradation (Table 4), which are involved in Carbohydrate metabolism, Folding, sorting and degradation, Glycan biosynthesis and metabolism, Metabolism of other amino acids, and Protein families: metabolism, indicating that *B. mycoides* NS75 has the ability to degrade proteins in tobacco leaves. During the fermentation process of tobacco leaves, *B. mycoides* NS75 can degrade proteins and other macromolecular substances in tobacco leaves to produce amino acids and other degradation products. In the subsequent heating and drying process of tobacco leaves, amino acids in tobacco leaves can serve as reaction substrates to produce pleasant aromas through the Maillard reaction, significantly improving the quality defects of tobacco leaves and enhancing the industrial usability of tobacco leaves (Zhou et al., 2020).

Through in-depth analysis of the whole genomes of the endophytic bacilli *B. halotolerans* NS36 and *B. mycoides* NS75, we identified potential key genes in enhancing the sensory quality of tobacco leaves. Although both strains can enhance the sensory quality of tobacco leaves, they may affect the chemical composition of tobacco leaves through different mechanisms. Specifically, *B. halotolerans* NS36 mainly secretes enzymatic systems related to the degradation of lignin, cellulose, starch, and pectin, reducing irritant substances in tobacco leaves, thereby reducing unpleasant tastes and making the sensory quality more balanced. *B. mycoides* NS75 secretes enzymatic systems related to glycoside hydrolysis and proteins, increasing the content of glycosidic aglycones and Maillard reaction products such as flavor substances in tobacco leaves, thereby enhancing the aroma quality and quantity of tobacco leaves. To verify the degradation capabilities of these strains, we conducted *in vitro* enzyme activity assays to support the results of the whole-genome analysis. *B. halotolerans* NS36 showed degradation capabilities against lignin, cellulose, starch, and pectin, while *B. mycoides* NS75 showed effective degradation against proteins and *β*-glucosides (Figure 4C).

## 5 Conclusion

This study isolated 11 endophytic bacteria from fresh tobacco leaves, with particular attention given to *Bacillus halotolerans* NS36 and *Bacillus mycoides* NS75, as they demonstrated significant effects on enhancing the sensory quality of tobacco leaves during the fermentation process. Chemical composition analysis and whole-genome sequencing revealed that these two strains improve tobacco quality through different mechanisms. *B. halotolerans* NS36 primarily secretes enzymatic systems related to the degradation of lignin, cellulose, starch, and pectin, reducing irritants in tobacco leaves, thereby decreasing unpleasant tastes and balancing sensory quality. *B. mycoides* NS75, on the other hand, secretes enzymatic systems related to glycoside hydrolysis and proteins, increasing the content of glycosidic aglycones and Maillard reaction products, which are aromatic substances in tobacco leaves, thus enhancing the aroma quality and quantity. *In vitro* enzyme activity assays further confirmed the degradation capabilities of these strains. Based on the aforementioned research findings, we believe that the enzymes produced by endophytic bacilli during the tobacco fermentation process play an important role in improving the aroma of tobacco leaves and reducing irritation, thereby enhancing the overall sensory quality of tobacco leaves.

The findings of this study provide a new perspective for the tobacco industry. By using endophytic bacilli, it is possible to improve the off-flavors of tobacco leaves and enhance the aroma without increasing harmful substances. For example, *B. halotolerans* NS36, due to its high efficiency in the degradation of lignin, cellulose, starch, and pectin, is suitable for fermenting tobacco leaves with high irritant substance content; while *B. mycoides* NS75, due to its expertise in the hydrolysis of proteins and glycosidic substances, is more suitable for enhancing the aroma quality of tobacco leaves. This customized fermentation strategy is expected not only to improve the industrial applicability of tobacco leaves but also to enhance the market competitiveness of products. In addition, through gene editing technology, the expression of these strains’ enzymes can be further optimized to meet different fermentation needs of tobacco leaves, customizing personalized treatment plans for different varieties and grades of tobacco leaves. This can not only improve the overall quality of tobacco leaves but also bring a richer and more diverse sensory experience to tobacco products, meeting the needs of different consumer groups.

## Data Availability

The datasets presented in this study can be found in online repositories. The names of the repository/repositories and accession number(s) can be found in the article/Supplementary material.
