# Supplementary material for: Influence of specific tobacco endophytic Bacillus on tobacco leaf quality enhancement during fermentation
Source: Front Microbiol. 2024 Nov 25;15:1468492. doi: 10.3389/fmicb.2024.1468492 (PMC11625772; doi:10.3389/fmicb.2024.1468492)
Supplement: Supplementary file 1 [file Data_Sheet_1.pdf]

Supplementary Table S1. Sensory quality assessment Scoring Table for Tobacco Fermented by 11 Strains of Endophytic Bacteria.

sample	increasing aroma				decreasing offensive taste				total score
	aroma quality	aroma quantity	diffusivity	after taste	offensive taste	irritancy	smooth	cleanness	
CK	5.9	6.0	6.0	6.0	5.1	5.3	5.9	5.9	46.1
YC18	6.3	6.2	6.0	6.0	5.4	5.5	6.2	6.2	47.8
NS36	6.2	6.0	6.0	6.1	5.6	5.5	6.1	6.4	47.9
NS39	6.0	6.5	6.0	6.0	5.0	5.0	6.0	6.0	46.5
NS47	6.0	6.0	6.0	6.0	5.0	5.0	6.0	6.0	46.0
NS51	6.5	6.0	6.0	6.2	5.1	5.3	6.2	6.3	47.6
NS55	6.0	6.0	6.0	6.0	4.7	5.0	6.0	6.0	45.7
NS59	6.0	6.0	6.0	6.0	4.6	4.5	6.0	6.0	45.1
NS63	5.5	6.0	6.0	6.0	5.0	5.6	6.1	6.1	46.3
NS65	5.5	6.0	6.0	6.0	5.2	5.0	6.0	6.0	45.7
NS71	5.9	6.0	6.0	6.0	5.1	5.3	5.9	5.9	46.1
NS75	6.6	6.6	6.3	6.1	5.1	5.3	6.1	6.1	48.1
NS76	6.5	6.5	6.0	6.0	5.1	5.0	6.0	6.0	47.1

Note: aroma quality: the quality of the aroma; aroma quantity: the richness of the aroma; diffusivity: the degree of exposure of aroma; after taste: the taste sensation left behind after exhaling smoke; offensive taste: inconsistent and uncomfortable breath; irritancy: the discomfort caused by smoke to the senses; smooth: particle feel and smoothness of smoke particles; cleanness: the feeling of cleanliness and no residue in all parts of the mouth.

Supplementary Table S2. Detection of 17 Amadori compounds in tobacco leaves based on near-infrared spectroscopy.

Sample	Glu-An	Fru-Amb	Fru-His	Fru-Pro	Fru-Val	Fru-Thr	Fru-Gly	Fru-Ala	Fru-Asn	Fru-Asp	Fru-Gln	Fru-Glu	Fru-Ile	Fru-Leu	Fru-Tyr	Fru-Phe	Fru-Trp
CK	242	2123	107	9867	212	27	25	1768	4177	1157	2431	932	23	48	125	1086	490
NS36	264	2104	111	9901	210	26	24	1834	4165	1192	2480	1000	23	50	125	1090	489
NS75	293	2123	115	10099	216	26	26	1848	4167	1145	2535	1060	24	53	125	1098	488

Note: Glu-An: Glucosamine; Fru-Amb: 1-deoxy-1-L-aspartyl-D-fructose; Fru-His: N-(1-deoxy-β-d-fructopyranos-1-yl)-l-histidine; Fru-Pro: N-(1-deoxy-β-d-fructopyranos-1-yl)-l-proline; Fru-Val: N-(1-deoxy-β-d-fructopyranos-1-yl)-l-valine; Fru-Thr: N-(1-deoxy-β-d-fructopyranos-1-yl)-l-threonine; Fru-Gly: N-(1-deoxy-β-d-fructopyranos-1-yl)-l-glycine; Fru-Ala: N-(1-deoxy-β-d-fructopyranos-1-yl)-l-alanine; Fru-Asn: N-(1-deoxy-β-d-fructopyranos-1-yl)-l-asparagine; Fru-Asp: N-(1-deoxy-β-d-fructopyranos-1-yl)-l-aspartic acid; Fru-Gln: N-(1-deoxy-β-d-fructopyranos-1-yl)-l-glutamine; Fru-Glu: N-(1-deoxy-β-d-fructopyranos-1-yl)-l-glutamic acid; Fru-Ile: N-(1-deoxy-β-d-fructopyranos-1-yl)-l-isoleucine; Fru-Leu: N-(1-deoxy-β-d-fructopyranos-1-yl)-l-leucine; Fru-Tyr: N-(1-deoxy-β-d-fructopyranos-1-yl)-l-tyrosine; Fru-Phe: N-(1-deoxy-β-d-fructopyranos-1-yl)-l-phenylalanine; Fru-Trp: N-(1-deoxy-β-d-fructopyranos-1-yl)-l-tryptophan.

Supplementary Table S3. Detection results of 55 aromatic components in tobacco leaves.

compounds	CK-1	CK-2	CK-3	NS36-1	NS36-2	NS36-3	NS75-1	NS75-2	NS75-3
furfuryl alcohol	2.4827	2.5712	2.5571	2.7684	2.5103	2.6680	2.7174	2.8605	2.7651
benzylalcohol	5.7350	5.6780	5.7067	5.9899	5.6738	6.9175	7.4489	7.3306	7.3399
phenylethanol	4.9432	5.0248	4.9851	5.0097	5.1587	5.1134	5.4112	5.5298	5.4617
nerolidol	0.1821	0.1879	0.1839	0.1794	0.1783	0.1848	0.1945	0.1990	0.1900
2(5H)-furanone	4.3094	4.3201	4.3526	4.6532	4.7815	4.7838	4.6731	4.7729	4.9607
2-hydroxycyclopent-2-en-1-one	2.8909	2.9047	2.8515	3.0438	2.9916	2.8916	4.0091	3.6929	3.7117
3-methyl-2-cyclopenten-1-one	0.1320	0.1321	0.1301	0.1233	0.1325	0.1235	0.1330	0.1378	0.1365
6-methylhept-5-en-2-one	0.8930	0.8323	0.7766	0.8800	0.9326	0.9388	0.9119	0.7735	0.7938
methyl cyclopentenolone	0.6829	0.7750	0.7772	0.8175	0.7116	0.7067	0.8422	0.7679	0.8000
4-methyl-2(5H)-furanone	0.5273	0.6137	0.5597	0.5962	0.6515	0.6956	0.7521	0.7514	0.7114
acetophenone	0.2458	0.2086	0.2570	0.2226	0.2221	0.2183	0.2302	0.2042	0.3112
furfuryl hydroxymethyl ketone	1.9574	2.0863	2.0988	2.2483	2.2588	2.1175	2.4406	2.3038	2.5778
1,4-cyclohexanedione	0.1212	0.1426	0.1313	0.1438	0.1494	0.1684	0.1966	0.2026	0.2239
6-methyl-3,5-heptadien-2-one	0.3963	0.3938	0.4015	0.3945	0.3925	0.3939	0.4006	0.3895	0.4287
isophorone	0.1787	0.1838	0.1731	0.1791	0.1843	0.1783	0.1885	0.1891	0.1991
4-oxoisophorone	0.4288	0.4609	0.4518	0.4121	0.4298	0.4333	0.4066	0.4049	0.4573
methylacetophenone	1.0946	1.0043	1.0235	0.9379	0.9798	0.9963	0.9706	0.8944	1.0904
β-damascenone	1.0489	1.0396	0.9213	0.9544	0.9876	0.9158	1.2718	1.1114	1.0634
damascone	1.0646	1.0678	0.9643	0.9806	1.0304	1.0053	1.0266	0.9767	1.0764
geranylacetone	2.2655	2.2552	2.2863	2.3593	2.5672	2.4943	2.6245	2.6839	2.6380
β-Ionone	0.4597	0.4826	0.4895	0.4541	0.5194	0.4970	0.5058	0.4491	0.4747
farnesylacetone	3.7547	3.6576	3.7281	3.8646	3.8684	3.7396	3.9069	4.0706	3.9977
furfural	2.0715	2.0758	1.8994	1.9768	2.0454	1.9117	1.9349	1.9566	2.0172
5-methylfurfural	0.8044	0.9859	0.7054	0.7537	0.7344	0.7220	0.7542	0.6643	0.7613
benzaldehyde	1.8984	1.9445	1.9180	2.1902	2.5050	2.4179	2.3074	2.1582	2.1996
phenylacetaldehyde	2.4015	2.2265	2.1360	2.3780	2.0804	2.5508	2.5296	2.3589	2.2652
3-methylbenzaldehyde	0.3667	0.3345	0.3221	0.3271	0.3532	0.3188	0.3451	0.3035	0.4426
saffron aldehyde	0.4135	0.4310	0.3873	0.3830	0.4319	0.3969	0.4240	0.3842	0.4687
β-cyclocitral	0.3306	0.3020	0.3089	0.2792	0.3252	0.3116	0.3148	0.2588	0.3250
5-hydroxymethylfurfural	15.5594	16.6878	15.1529	17.8449	19.0353	18.5879	17.7155	18.6946	19.0070
vanillin	1.9409	1.8923	2.0223	2.0689	2.3970	2.3927	2.5117	2.6005	2.8318
4-ethyl-2-methoxyphenol	0.0166	0.0194	0.0157	0.0237	0.0253	0.0243	0.0170	0.0159	0.0196
4-Hydroxy-3-methoxystyrene	19.3702	19.3696	19.4246	24.2959	25.5772	27.5112	21.0357	23.0206	23.3859
4-acryloyl-2-methoxyphenol	0.7548	0.7164	0.7565	0.8316	0.8691	0.8300	1.0120	0.9747	1.0607
2,6-dimethoxyphenol	0.6843	0.7088	0.6981	0.7586	0.7498	0.7637	0.7018	0.7110	0.7193
2-Methyl-1-butanol	0	0	0	0	0	0	0	0	0
2-Ethylpyridine	0	0	0	0	0	0	0	0	0
2,5-Dimethylpyridine	0	0	0	0	0	0	0	0	0
2-Methyl-2-cyclopenten-1-one	0	0	0	0	0	0	0	0	0
3-Ethylpyridine	0	0	0	0	0	0	0	0	0
2-Cyclohexen-1-one	0	0	0	0	0	0	0	0	0
2-Hydroxy-γ-butyrolactone	0	0	0	0	0	0	0	0	0

2,3-Dimethyl-2-cyclopenten-1-one	0	0	0	0	0	0	0	0	0
2-Methylphenol	0	0	0	0	0	0	0	0	0
Pineapple ketone	0	0	0	0	0	0	0	0	0
3,4-Dimethyl-1,2-cyclopentanedione	0	0	0	0	0	0	0	0	0
n-Octanol	0	0	0	0	0	0	0	0	0
Linalool	0	0	0	0	0	0	0	0	0
3-Acetoxypyridine	0	0	0	0	0	0	0	0	0
Ethyl cyclopentenolone	0	0	0	0	0	0	0	0	0
3,4-Dimethylphenol	0	0	0	0	0	0	0	0	0
2,4,6-Trimethylphenol	0	0	0	0	0	0	0	0	0
Citronellol	0	0	0	0	0	0	0	0	0
Geraniol	0	0	0	0	0	0	0	0	0
3-Hydroxybenzaldehyde	0	0	0	0	0	0	0	0	0

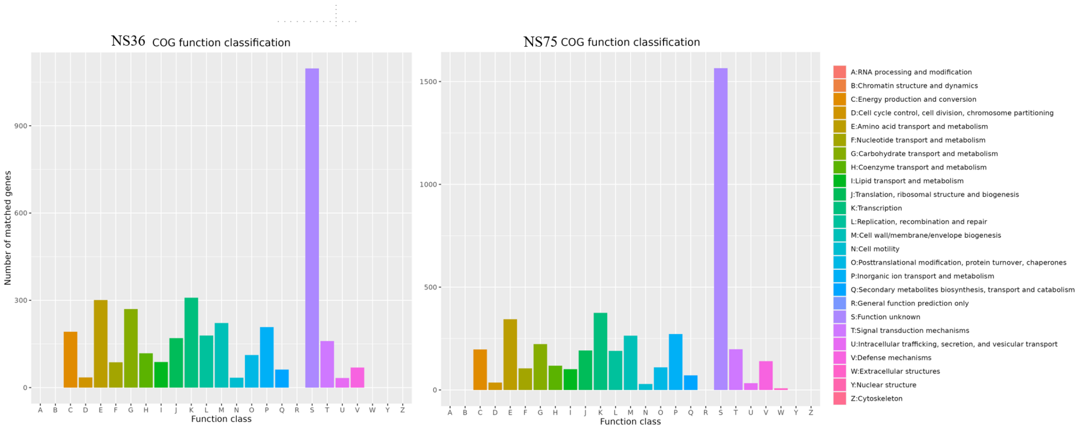

Supplementary Figure S1. Endogenous bacterial eggNOG functional classification diagram.

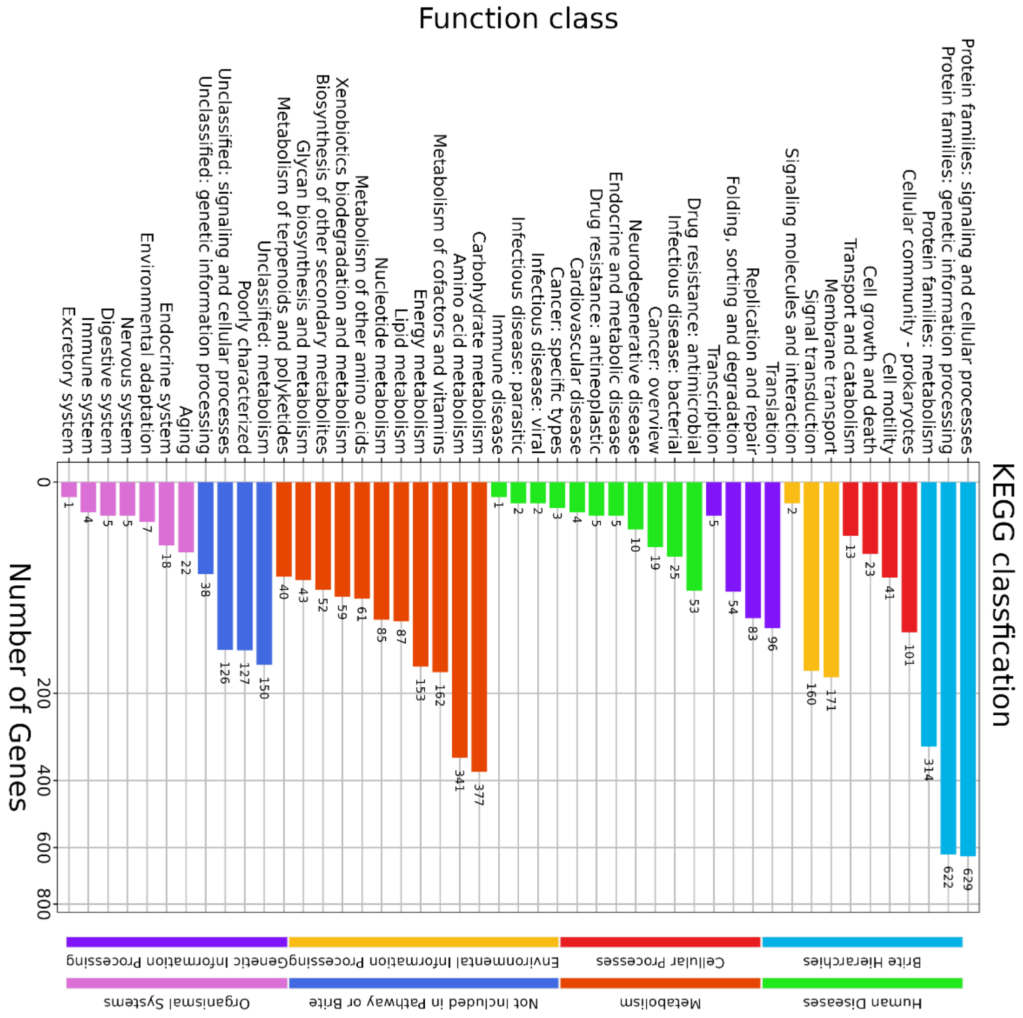

Supplementary Figure S2. KEGG annotation of the B. mycoides NS75 protein-coding gene.
